# Military Preparedness

**Published:** 1916-12

**Authors:** 


**A Monthly Review of Medicine and Surgery**

EDITOR AND PUBLISHER, DR. A. L. BENEDICT, 228 Summer St., corner of Elmwood Ave., Buffalo, (Address for all communications. Please make personal and telephone calls before 1 P. M.)

Third, in age, on the western continent. Independent, but supports professional organizations. News limited mainly to Western N. Y. and Penn.

Subscription $2.00 a year; $3.00 for two years in advance. Changes and errors in address or failure or duplication of delivery, should be reported immediately.

Yearly Volume 72 DECEMBER, 1916 Number 5

### Military Preparedness.

Various quantitative estimates in the discussion of preparedness suggest a point that ought to be obvious but that seems to have been almost universally overlooked. Luxembourg is a good deal better off than Belgium.

